# Neighborhood Availability and Use of Food, Physical Activity, and Social Services Facilities in Relation to Overweight and Obesity in Children and Adolescents

**DOI:** 10.1177/03795721221146215

**Published:** 2023-01-04

**Authors:** Camila Elizandra Rossi, Maria Gabriela M. Pinho, Elizabeth Nappi Corrêa, Ângelo Horta de Abreu, Cassiano Ricardo Rech, Jorge Ricardo da Costa Ferreira, Francisco de Assis Guedes de Vasconcelos

**Affiliations:** 1 Universidade Federal da Fronteira Sul, Realeza—Paraná, Brazil.; 2 Amsterdam Public Health Research Institute, Amsterdam UMC, Vrije Universiteit Amsterdam, The Netherlands; 3 Upstream Team, Amsterdam UMC, Vrije Universiteit Amsterdam, The Netherlands; 4 Universidade Federal de Santa Catarina. Rua Engenheiro Agronômico Andrei Cristian Ferreira, s/n—Centro de Ciências da Saúde, Trindade, Florianópolis—Santa Catarina, Brazil.; 5 Gis Especialist. Imagem—Enterprise for Geographic Intelligence Solutions. Belo Horizonte—Minas Gerais, Brazil.; 6 Programa de Pós-Graduação em Educação Física (PPGEF), Campus Universitário Trindade, Florianópolis—Santa Catarina, Brazil.; 7 Universidade Nova de Lisboa, Faculdade de Ciências Sociais e Humanas. Lisbon, Portugal.

**Keywords:** built environment, social environment, physical activity environment, obesity, youth

## Abstract

**Objective:**

To evaluate the association of a combined measure of availability and use of facilities from the food environment and overweight (including obesity) among schoolchildren, while taking into account the physical activity and social-assistance environments.

**Methods:**

Cross-sectional study with a probabilistic sample of schoolchildren aged 7 to 14 years living in a southern Brazilian city (n = 2026). Multilevel analyses were performed with overweight as outcome and the food environment as main exposure. Models were adjusted for the physical activity and social-assistance environments, as well as individual and other residential neighborhood characteristics.

**Results:**

Greater availability of restaurants around the home was associated with higher odds of overweight (odds ratio [OR] = 1.40; 95% CI = 1.06-1.85). Stronger associations were found for schoolchildren reporting to use restaurants (OR = 1.48; 95% CI = 1.15-1.90). This association remained significant after adjusting for the presence of other food retailers. Schoolchildren who had social-assistance facilities around their homes, but reported not to use them, showed consistently higher odds of being overweight (OR = 1.34; 95% CI = 1.01-1.78) as compared to schoolchildren who had these facilities near home and used them. The physical activity environment was not associated with the outcome.

**Conclusions:**

Availability and use of the food and social-assistance environments were significantly associated with overweight (including obesity) among the schoolchildren. Future research should consider the use of environmental facilities in combination to their geographical availability. Our results highlight the need for policies that limit the access to obesogenic food outlets by children and adolescents.

## Introduction

Most studies on determinants of childhood obesity have focused on individual level determinants aiming to inform small-scale interventions, such as school-based interventions.^1,2^ Although many of these school-based studies have shown positive results toward weight loss and improving dietary habits and physical activity, these interventions are limited to small settings and reach a limited number of children. Thus, there is a need for upstream level interventions that have the potential for behavior change on a wider scale.^3^ Indeed, research interest is shifting toward generating evidence to inform upstream level interventions (eg, policies and regulations). In this context, research on environmental level determinants of childhood obesity has been increasingly popular in the past decades.^4-6^ Many of these studies have focused on the food environment, as defined by the presence (around the home or school) of food outlets such as grocery stores, snack bars, and restaurants.^7-11^ Evaluating the food environment is highly relevant in Brazil, because the prevalence of out-of-home food intake reached 40.2% and 36.5% of adults in the years 2008 to 2009 and 2017 to 2018, respectively. The most frequent items bought away from home in both periods were mostly unhealthy food such as fried and baked snacks, soft drinks, pizza, sweets, and sandwiches.^12,13^ However, evidence from systematic reviews is mixed (ie, both negative and positive relationships are found) and they provide limited evidence for an association between the food environment and body weight.^10,11^ It is also important to note that the major part of the studies found by systematic reviews is cross-sectional, while only 10 were longitudinal.^10,11^ It has been suggested that future longitudinal studies are needed to account for potential changes in the food environment over time, which may improve the quality of the evidence base and provide more consistent findings.^10^

One potential explanation for these mixed findings is the underlying assumption that the geographical availability of a food outlet translates to the use of that outlet. As most studies to date rely on this assumption, current evidence may be biased, reflecting a gap between the assumed and actual exposure of individuals to the food environment. Indeed, research has found more consistent associations when considering exposure to the perceived environment.^14,15^ The perceived environment may be defined as the individual perception about the presence of facilities available around their home, thus the perceived environment has been used as a proxy for the use of neighborhood facilities. For example, research from Brazil has found that both the availability and use of restaurants in the residential neighborhood were positively associated with overweight and obesity in schoolchildren.^16^ Therefore, more research is needed to investigate not only the geographical availability of food outlets but also their use in relation to obesity.

Another gap in food environment research is the predominant focus on the food environment in isolation. That is, many studies define exposure to only one type of food outlet, ignoring the potential influence of other food outlet types on the association being analyzed^17-19^ and also ignoring the presence of other important facilities from the built and social environments. Indeed, there is evidence for an inter-related influence of different environmental constructs on individuals’ health.^20-23^ That is, not a single neighborhood construct would be enough to explain the influence of environmental characteristics and health outcomes. Some efforts have been made to analyze different constructs of the environment in relation to obesity, for example, by combining the influence of the social environment as defined by social capital, presence of assistance facilities, and social ties and crime; and the physical activity environment, as defined by the presence of bike paths and sport courts.^21,24-26^ In addition, previous longitudinal evidence focusing on the social and physical activity environments shows that an increase in the number of outdoor recreational facilities, community centers, and associations in a buffer of 5 km around US homes was associated with lower the odds of obesity when the evaluated adolescents became adults.^21^ Likewise, the absence of bike paths and living in higher density areas were associated with a higher likelihood of being overweight and obese in Chinese children.^22^ Evidence from Brazil suggests that higher perceived availability of facilities of the food, physical activity, and social-assistance environments near home were associated with higher body mass index (BMI) in schoolchildren from low-income families.^23,27^ However, current evidence mainly focuses on a single construct, for example, only the food or the physical activity environment.^10,27,28^ Therefore, more studies are needed to explore the combined influence of the mentioned constructs of the environment in relation to childhood obesity in different settings.

The aim of this study was to investigate the association between availability and use of the food, physical activity, and social environments and overweight and obesity among schoolchildren aged 7 to 14 years living in the city of Florianópolis (South of Brazil).

## Methods

### Study Design, Sampling, and Data Collection

Florianópolis is a coastal city and the capital of the Brazilian state of Santa Catarina, located in the country’s south administrative region. In 2013, the prevalence of overweight/obesity in Florianópolis was 36.7% among younger children (7-10 years old) and 29.8% among adolescents (11-14 years old).^29^ In 2010, Florianópolis’ Index of Human Development (IHD) was classified as very high (0.847), while for Brazil it was 0.727.^30^ The IHD is a composite measure of a city or country’s longevity, education, and income. Despite its high IHD, the city still presents social inequalities, showing a Gini Index of 0.547 (the closer to 1, the greater the social inequalities between residents). The Gini Index shows the proportion of the population that receives from the lowest to highest incomes among those on the bottom of income,^31^ and Florianópolis’ Gini Index, although lower than the value found for Brazil (0.6086), is not above the recommended (< 0.500). In the southern region of Brazil, where Florianopolis is located, the percentage of food insecure families varies from 6.0% (severe food insecurity) to 32.3% (mild food insecurity), although data from Florianopolis are not known.^32^

In this cross-sectional analysis, data from 2484 children and adolescents enrolled at public or private schools in Florianópolis were used. Data were collected in 2013 as part of a larger survey called “Study of the Prevalence of Obesity in Children and Adolescents” in Florianópolis, SC (EPOCA). To estimate the sample size, the following parameters were used: expected prevalence of overweight/obesity of 38% (based on the previous waves); total number of schoolchildren aged 7 to 14 years in the municipality (n = 45 247); sampling error of 3.5 percentage points (2-tailed) and 95% CI, resulting in an initial sample size of 727 students. Considering a design effect of 1.8, the sample size needed would be 1309 schoolchildren. Considering the stratification by age-group (7-10 years and 11-14 years), the sample size was doubled, totalizing 2618 children to be evaluated. Adding 10% to this value due to possible losses or refusals, the final sample size was estimated to be 2880 schoolchildren.^29,33,34^ The reached sample size is sufficient to find odds ratio (OR) higher than 1.5 with a power of 80%, and an α-error of 5%. The sample was selected by clusters and according to the number of students enrolled in each school. This procedure aimed to ensure that the sample was representative of the regions in which the population lives in terms of residential density and income. The sampling method has been described in greater detail elsewhere.^29,34^ From this sample, 4.2% (n = 104) did not live in Florianópolis; 4.7% (n = 117) did not inform their residential addresses; 1.5% (n = 37) were not found in the geocoding location; and 0.8% (n = 20) did not have valid data on the outcome measure. The total number of schoolchildren included in this analysis was 2206 (88.8% of the initial sample investigated). This study was conducted according to the Declaration of Helsinki guidelines, all the procedures involving study participants were approved by the Human Research Ethics Committee at Federal University of Santa Catarina (UFSC), under review process no. 120341/2012. Written informed consent was obtained from all participants.

### Measures

#### Outcome measure

The anthropometric measurements (weight and height) were collected by trained researchers and following the assessment protocol of the International Society for the Advancement of Kinanthropometry.^35^ The intra- and interexaminer technical errors of measurement were calculated (coefficient *R* ≥ 0.92) and were considered satisfactory for the fieldwork.^36^ To calculate the prevalence of overweight and obesity, cutoff points were determined as BMI/age and sex ≥ + 1 *z* scores for overweight (including obesity), according to the World Health Organization reference curves.^37^ Only 1.5% of children were underweight, therefore those were included in the category “not overweight,” as well as the children with a healthy weight. Both children classified as overweight and obese were included in the category “overweight (including obesity),” according to the terminology used by Cole et al.^38,39^ We chose to analyze overweight and obesity together because this is a commonly used approach in nutritional epidemiology and because our power calculations were made based on prevalence of overweight/obesity combined.^40-42^ For the sake of simplicity, this study will refer to the latter category as “overweight.” As a way of compensating the students and school’s effort, at the end of the survey each participating school received an individualized report with their students’ assessments. Individually, each student also received his/her assessment.

A pilot study was also carried out at a school that was not included in the final sample of this research, aiming at the adaptation of the instruments and establishment of the fieldwork routine. This pilot study included 30 schoolchildren from 7 to 14 years of age enrolled in a school that did not take part in the research. The administration of the questionnaires and collection of anthropometric measures were conducted by the same research team that performed data collection in the original study and following the predefined research protocol. The pilot survey aimed at the parents was sent via school communication followed by a phone interview to check the understanding of the questions and to estimate the time to complete the questionnaire.

#### Exposure measures

##### Use of facilities of the food, social-assistance, and physical activity environments

All schoolchildren aged 7 to 14 years enrolled in the selected schools were invited to participate. Parents who gave their consent were invited to answer a self-administered survey that was sent via regular school communication. To construct the data collection questionnaire, especially survey questions related to the use of facilities from the food, social-assistance, and physical activity environments, we performed an extensive literature research to identify relevant tools.^43-46^ To translate and adapt these tools, which were originally published in English, we used a “collaborative and iterative translation” approach, a technique that prevents literal translations resulting instead, in a translation that is meaningful for the respondents.^47^ Thus, the questions were first translated from English to Portuguese, after that, the translations were evaluated by experts in order to reach agreement on meaning and correspondence. Then, the adapted instruments were pilot-tested in a school that was not included in the sample, aiming at the amelioration of the questions, as explained above. Regarding the food environment, parents were asked how often in the past 6 months their children visited different types of food outlets, including restaurants, street food vendors, snack bars, and fast food outlets; and in which type of facilities the family currently acquire food items for cooking at home, including bakeries, supermarkets, and mini markets. Regarding the physical activity environment, parents were asked how often in the past 6 months their children used different types of outdoor recreational facilities for physical activity including parks/playgrounds, sports courts, football pitches, skate parks, and open-air gyms (ie, community areas with exercise equipment). Response options for these 2 domains of the environment were “never,” “rarely” (2 or 3 times a year), “weekly,” “twice a month,” or “monthly,” except for the use of bakeries, mini markets, and supermarkets which was originally asked in 2 categories (“did not use” and “used”).^48,49^ Based on the data distribution and due to the intention of creating one combined variable for “use of facilities” and for “geographical availability of facilities,” the reported frequency of use of all facilities of the food and physical activity environments was categorized into 2 categories did not use (never and rarely) and used (weekly, twice a month, and monthly).

In Brazil, social-assistance services are actions promoted by governmental and nongovernmental institutions aiming at securing citizen’s basic needs. The social-assistance environment was evaluated using 4 types of facilities related to both social assistance and health care: community health care centers; centers for Social Assistance (CSA); an instructional facility named “Centers for Supplementary Education, which is an after school program; and neighborhood associations. Such facilities are generally located in socially deprived areas, and their use is free of charge.^50^ Participation in cash transfer and food transfer programs, that are Brazilian policies executed by the CSA to attend families in social vulnerability, was also evaluated (see Table 1 for details). Parents were asked whether or not their children benefited from any of these social services in the past 6 months.

**Table 1.** Description and Classification of Analyzed Facilities From Food, Physical Activity, and Social-Assistance Environments.

Analytical category	Facilities evaluated	Definition of the facility
Food environment^a^		
A. Restaurants	Full-service restaurants	Activities of selling and offering a-la-carte meals and table service. It usually opens only in the evening.
	Buffet lunch-restaurants	Provision of meals served on a buffet system, selling ready-to-eat meals in both self-service and by weight-service. It usually opens during lunch hours. Normally located near workplaces.
B. Snack outlets	Street food vendors	Retailers providing ready-to-eat food served on fixed or itinerant places as trailers, food trucks, or ambulant vendors.
	Bakeries	Retailers providing breads, cakes, cookies, and pastries. Fabrication can be local or provided by other food outlets. Table service is possible but is not the main store activity.
	Snack bars and fast food outlets	Retailers offering ready-to-eat food mainly consumed inside, including snacks, fast food (both fast-food chains and small local-owned fast food outlets), ice cream, tea, juices, sweetened beverages, and pastries. Fabrication can be local or provided by other food outlets. Drinks are only provided in combination with food.
C. Grocery stores	Supermarkets	Retailers providing diverse food products, including other type of products like domestic utilities, cleaning and personal hygiene products, and clothes. The selling area is bigger than 300 m^2^.
	Mini markets	Retailers providing mainly diverse food products, with a selling area smaller than 300 m^2^.
Physical activity environment		
Outdoor recreational facilities	Parks/playgrounds	Public open spaces including parks, local playgrounds, and ecological reserves that offer opportunity for leisure and sports.
	Sports courts	POS for individual or collective sports beyond soccer, as basketball, volleyball, handball, tennis, and others.
	Football pitches	POS composed by natural or synthetic grass, sand, or concrete ground.
	Skate parks	Spaces used solely by skaters, including public and open or private spaces.
	Open-air gyms	POS structured with equipments for individual exercise. Such spaces are placed and maintained by the municipality of Florianópolis as part of project to promote physical activity.
Social-assistance environment		
Social-assistance services facilities	Community health care centers	Public primary health care centers that are part of the National Brazilian Health System (SUS).
	Centers for Supplementary Education	Instructional facility offering educational, social, and artistic activities, complementary to formal education.
	Neighborhood association	Places where members of a neighborhood association meet to socialize and discuss community issues.
	Centers for Social-Assistance	Centers for the registration and assistance of families in social vulnerability. Responsible for the execution of several government programs as listed below.
	*Bolsa Família* Program	National cash transfer program targeted to families in social vulnerability, executed by the CSA.
	*Brasil Carinhoso* Program	National program for supplementation of nutrients as iron and Vitamin A, executed by the CSA.
	*Hora de Comer* Program	Municipal food transfer program targeting undernourished children, executed by the CSA.
	*Cesta Básica* Program	National food transfer program to families in social vulnerability, executed by the CSA.
	Social projects	Philanthropy and voluntary initiatives for social assistance, executed by nongovernmental organizations.

Abbreviations: CSA, centers for Social Assistance; IBGE, Brazilian Institute of Geographic and Statistics; POS, public open spaces.

^a^ Source of information: Wagner et al^29^ and IBGE (available at https://cnae.ibge.gov.br/?option=com_cnae&view=estrutura&Itemid=6160&chave=&tipo=cnae&versao_classe=7.0.0&versao_subclasse=9.1.0).

The classification of facilities of the food environment was based on the patterns of food purchased by Brazilian families living in the south region of Brazil evaluated on the Brazilian Acquisition Research data (Pesquisa de Orçamentos Familiares) from the Brazilian Institute of Geographic and Statistics (IBGE).^51^ Information on the use of different facilities of the food environment was summed and grouped into 3 food outlet groups, which were named as follows: (1) snack outlets (including snack bars, fast food outlets, street food vendors, and bakeries), (2) grocery stores (including mini markets and supermarkets), and (3) restaurants (including lunch restaurants that sell buffet meals by weight and on a buffet system, and *a la carte* restaurants). Schoolchildren and families who reported the use of at least one of the facilities into each group were considered “using the facility” (vs “not using”). All physical activity and social-assistance services facilities cited above were summed together to create the variables “use of recreational outdoor facilities” and “use of social-assistance services facilities.” Responses were dichotomized into “using” (if schoolchildren used at least one of the facilities into each domain of the environment) versus “not using” the facilities. Table 1 shows detail on the types and description of the evaluated facilities and their categorization as used in the statistical analyses.

##### Geographical availability of facilities of the food, social-assistance, and physical activity environments

Secondary data on geographical availability of food outlets in the city of Florianópolis were obtained from registers of the Municipal Department of Health. This surveillance database listed the food outlets available in the year 2013, including information on the name, address (street, neighborhood, and ZIP code), and type of establishment as defined by the municipality of Florianópolis. Detailed description of the procedures for data collection is described elsewhere.^16,52^ In summary, assuming that the municipal registers could be incomplete, data were triangulated from 5 other sources to check, and possibly update, the original database. Other sources of data included (1) printed and online directories listing commercial business (yellow pages); (2) consultation of a municipal decree that regulates street food vending^53^; (3) consultation of the list of municipal street markets provided by the Executive Department of Public Services of Florianópolis; (4) consultation of documents from the Brazilian Association of Bars and Restaurants; and (5) and official websites of fast food chains and other food vendors.^52^ More information on analyzed food outlets is found in Table 1.

Information on the social-assistance environment was also obtained from secondary data sources namely printed and online telephone directories and websites of governmental and nongovernmental organizations. Information on the social-assistance environment included address (street, neighborhood, and ZIP code) and type of establishment. Both databases on food and social-assistance environments were imported into QGIS software for geolocalization of addresses and ZIP codes. Google Earth and Street View tools were used aiming at greater spatial precision.

Regarding the physical activity environment, the availability of open and public places was collected via street audit, using a Garmin Global Navigation Satellite System receptor. Previous studies describe details of procedure collection and characteristics of the facilities.^54,55^ These data were geocoded using ArcGis (version 10.3.1).

The home address from each schoolchild was collected via questionnaire and geocoded using ArcGis version 10.3.1. After the geocoding of the facilities from the food, social-assistance, and physical activity environments, 400 and 800 m buffer zones around the children’s home were generated. These buffer sizes were chosen because they correspond to 5 and 10 walking minutes, respectively,^46,56,57^ and are commonly found in the literature.^10,58,59^

##### Operationalization of variables characterizing the availability and use of facilities of the food, social-assistance, and physical activity environments

The number of facilities within 400 and 800 m surrounding each schoolchild’s home for food, outdoor recreational facilities, and social-assistance services facilities was summed giving origin to discrete variables for each different type of facility. Due to the variable distribution and because of the intention of creating a combined variable for availability and use of facilities, each discrete variable was dichotomized within each buffer zone as follows: If at least one facility of the domain was present in each buffer, the facility was coded as “available”; if no facility was present as “not available.” A similar procedure was followed for the facilities of the food environment, where we analyzed 3 food outlet types: snack outlets, grocery stores, and restaurants. More details about the classification and definition of these facilities are provided in Table 1.

To create a variable representing geographical availability and use of facilities, a dummy variable was created combining each variable for the availability of facilities and the variables for use of facilities. This resulted in 3 variables reflecting availability and use of facilities of the food, social-assistance, and physical activity environments of which the categories are as follows: (1) “not available and not used,” (2) “available and not used,” and (3) “available and used.” Regarding the variables of the food environment, both snack outlets and grocery stores showed a low frequency for the category available and not used. Thus, this category was aggregated to the first one not available and not used, resulting in dichotomous variables (not available and/or not used or available and used). Restaurants, as well as “outdoor recreational facilities” and “social-assistance services facilities,” remained with 3 categories as there were no problems with the distribution of these variables.

##### Covariates

Sociodemographic characteristics were obtained from a questionnaire answered by the parents including information on children’s sex (male or female), age (continuous), and type of school (public or private). Maternal education, originally collected with 5 response options, was grouped into 3 categories due to variable distribution and to match the corresponding years for elementary, secondary, and higher education: 0 to 8 years; 9 to 11 years, and ≥ 12 years.

The city of Florianópolis is divided into 30 geographical areas, which are groups of census tracts in which results for demographic features exhibit statistical significance. Data on monthly income per household and number of inhabitants in each geographic area were collected from the 2010 demographic census.^60^ Taking into account the numbers of inhabitants and the territorial extension, we calculated the population density in km^2^ for each area using ArcGis. Participant’s addresses were linked to the correspondent area level income and population density, giving origin to the continuous variables “area level average income” and “area level population density.” Availability and use of the physical activity and social-assistance environments were used as covariates for statistical adjustment in the models.

### Statistical Analyses

The sample consisted of 2206 schoolchildren who had complete information on home addresses. The percentage of missing values on our variables ranged from 3% (maternal education) to 12% (use of snack outlets). Considering that missing values were assumed to be missing at random and that complete-case analyses may lead to biased results, we performed multiple imputation. Given our maximum percentage of missing values (12%), we imputed 10 data sets, as recommended by Rubin^61^ and Bodner.^62^ Descriptive statistics were performed on nonimputed data, while regression models were run on pooled estimates resulting from the 10 imputed data sets.

Descriptive statistics were performed on the entire sample and by weight status based on BMI. Differences between groups for qualitative variables were tested using χ^2^ tests, and differences among groups for continuous variables were tested using Mann-Whitney *U* tests. Independent models for availability and use of each food outlet were firstly run independently (models 1A, 1B, 2A, 2C, 3A, and 3B). Then, since food outlets often colocate and because the presence of different types of food outlets may simultaneously influence food behaviors,^24^ a fully adjusted model containing snack outlets, grocery stores, and restaurants together (models 4A and 4B) was also run. Models with the letters A and B present environmental variables derived within 400 and 800 m buffers, respectively. Spearman correlation analysis was performed to all exposure variables (Supplementary Table 2). The highest correlation coefficient found was 0.3823, thus, it can be concluded that the risk of multicollinearity in the analyzed models is low.

Clustering at the school level was tested by adding a random intercept for school to all models. Results from 2 log-likelihood tests showed significant clustering, and therefore multilevel logistic regression analyses were performed with participants (first level) clustered within school (second level). All models were a priori adjusted for the physical activity and social-assistance environments, as well as sociodemographic covariates (ie, age, sex, maternal educational, population density, and average area income). Descriptive and multilevel logistic regression analyses, as well as multiple imputations, were performed using Stata 13.0. A 5% significance level was adopted for hypothesis testing on bivariate analysis (*P* value ≤ .05). With regard to the multilevel logistic regression models, a significance association was determined when the confidence interval did not contain the value of 1.

## Results

**Figure 1.**
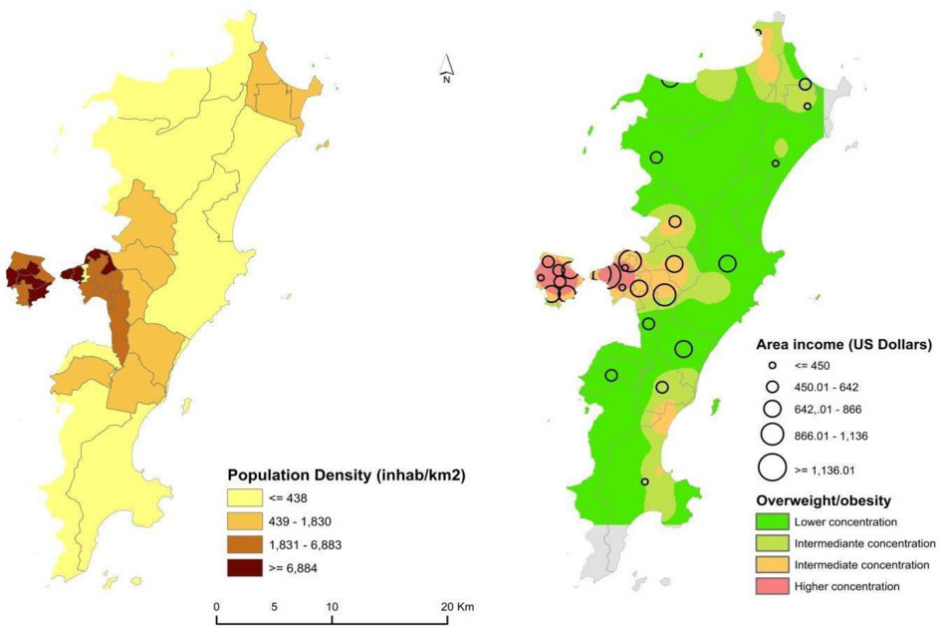
Family monthly income (in US dollars), absolute population, and absolute concentration of overweight—Florianópolis/SC, 2012/2013.

Table 2 shows the characteristics of the entire sample and stratified by weight status. Prevalence of overweight (including obesity) was 34.5% (21.5% of children presented overweight while 13% presented obesity), and median age was 10 years. Bivariate analysis showed that overweight was more frequent among boys in comparison to girls (*P* value < .001), among younger children in comparison to those older than 12 years (*P* value = .002), and among schoolchildren who had restaurants available around their home and use them versus those who do not have them available and/or not used them (*P* value = .001). Prevalence of overweight was also associated with the availability and use of social-assistance services facilities in both buffer zones as children with overweight more often had these facilities near home but did not use them, as compared to non-overweight school children in the same condition (*P* values = .005 for 400 m and .01 for 800 m). Figure 1 shows the geographical distribution of population density (left side) and the distribution of overweight, combined with area level income (right side), across the city of Florianópolis. On the right side of the figure, one can observe that the higher geographical concentration of cases of overweight coincides with areas of both lower and higher average income.

**Table 2.** Characteristics of 7- to 14-Year-Old Schoolchildren, Stratified by Weight Status, Florianópolis, Santa Catarina, Brazil, 2012/2013.

Variables	Categories	Total sample (n = 2206)		Not Overweight (n= 1473)		Overweight (n = 733)		*P* value^a^
		n	%	n	%	n	%	
Sex (n = 2206)	Female	1166	47.1	825	56.0	341	46.5	<**.001**
	Male	1040	52.9	648	44.0	392	53.5	
Age (median, IQR)		10, 8-12		10, 8-12		10, 8-11		**.002^b^**
Areal level population density (median, IQR) US$^c^		588.18, 131.81-2761.36		588.18, 131.81-2761.36		588.18, 159.09-3129.09		.09^b^
Area level average income (median, IQR)		966.81, 779.09-1366.36		966.81, 779.09-1366.36		952.27, 779.09-1366.36		.10^b^
Maternal education (years of study) (n = 2143)	0-8	582	27.2	400	28.1	182	25.3	.32
	9-11	750	35.0	486	34.1	264	36.7	
	≥12	811	37.8	538	37.8	273	38.0	
Availability and use of snack outlets—400 m (n = 1949)	Not available and/or not used	576	29.5	401	30.8	175	27.1	.09
	Available and used	1373	70.5	902	69.2	471	72.9	
Availability and use of grocery stores—400 m (n = 1913)	Not available and/or not used	508	26.6	355	27.7	153	24.2	.10
	Available and used	1405	73.4	927	72.3	478	75.8	
Availability and use of restaurants—400 m (n = 2125)	Not available and not used	588	27.7	429	30.2	159	22.5	**.001**
	Available and not used	532	25.0	345	**24.3**	187	**26.5**	
	Available and used	1005	47.3	645	**45.5**	360	**51.0**	
Availability and use of snack outlets—800 m (n = 1949)	Not available and/or not used	249	12.8	177	13.6	72	11.1	.12
	Available and used	1700	87.2	1126	86.4	574	88.9	
Availability and use of grocery stores—800 m (n = 1913)	Not available and/or not used	142	7.4	93	7.2	49	7.8	.68
	Available and used	1771	92.6	1189	92.3	582	92.2	
Availability and use of restaurants—800 m (n = 2125)	Not available and not used	150	7.1	108	7.6	42	6.0	.36
	Available and not used	706	33.2	470	33.1	236	33.4	
	Available and used	1269	59.7	841	59.3	428	60.6	
Availability and use of outdoor recreational facilities—400 m (n = 2206)	Not available and not used	1094	49.6	751	51.0	343	46.8	.17
	Available and not used	170	7.7	111	7.5	59	8.0	
	Available and used	942	42.7	611	41.5	331	45.2	
Availability and use of outdoor recreational facilities—800 m (n = 2206)	Not available and not used	465	21.1	333	22.6	132	18.0	**.03**
	Available and not used	318	14.4	204	**13.9**	114	**15.6**	
	Available and used	1423	64.5	936	63.5	487	66.4	
Availability and use of social-assistance facilities—400 m (n = 2206)	Not available and not used	1037	47.0	721	49.0	316	43.1	**.005**
	Available and not used	408	18.5	247	**16.7**	161	**22.0**	
	Available and used	761	34.5	505	**34.3**	256	**34.9**	
Availability and use of social-assistance facilities—800 m (n = 2206)	Not available and not used	500	22.7	360	24.4	140	19.1	**.01**
	Available and not used	589	26.7	374	**25.4**	215	**29.3**	
	Available and used	1117	50.6	739	**50.2**	378	**51.6**	

Abbreviation: IQR, interquartile range.

^a^ χ^2^ test of independence. *P* value criteria for significance is ≤ .05.

^b^ Mann-Whitney *U* test for nonparametric variables.

^c^ Data were reported in Brazilian Reais (R$) and transformed to US dollar (the value of the US dollar varied from R$2.03 to R$2.37 during the data collection period).

Table 3 shows coefficients from multilevel analyses regarding the independent association between availability and use of the food, physical activity, and social-assistance environments and overweight. Greater availability of restaurants within 400 m from home (model 3A), but not within 800 m (model 3B), was associated with overweight, with stronger coefficients for children who had restaurants available and used them. Children with a restaurant available but not using it had 40% higher odds of overweight as compared to children who did not have restaurants available (OR = 1.40; 95% CI = 1.07-1.84). Children with a restaurant available and reporting to use it had 48% higher odds of being overweight as compared to children who did not have restaurants available (OR = 1.48; 95% CI = 1.15-1.90) that translates into an 8% difference in the odds of obesity by having restaurants near home and using them as compared to having restaurants near home but not using them. No significant association was found for the other food outlets namely snack outlets (models 1A and 1B) and grocery stores (models 2A and 2B), neither for availability and use of outdoor recreational facilities (across all models).

**Table 3.** Multivariate Analyses of Association Between Overweight and One Type of Food Outlets, Outdoor Recreational Facilities, and Social-Assistance Features Among Schoolchildren Aged 7 to 14 Years Living in Florianópolis, SC, Brazil, 2012/2013.

Model 1: Availability and use of snack outlets					
		Model 1A^a^ Within 400 m from home		Model 1B^a^ Within 800 m from home	
		OR	95% CI	OR	95% CI
Availability and use of snack outlets	Not available and/or not used	1.00		1.00	
	Available and used	1.08	0.85-1.38	1.14	0.83-1.55
Availability and use of outdoor recreational facilities	Not available and not used	1.00		1.00	
	Available and not used	1.20	0.84-1.73	1.34	0.96-1.87
	Available and used	1.11	0.88-1.39	1.15	0.87-1.51
Availability and use of social-assistance facilities	Not available and not used	1.00		1.00	
	Available and not used	**1.42**	**1.07**-**1.87**	**1.35**	**1.00**-**1.82**
	Available and used	1.09	0.85-1.40	1.18	0.89-1.56
Model 2: Availability and use of grocery stores					
		Model 2A^a^ Within 400 m from home		Model 2B^a^ Within 800 m from home	
		OR	95% CI	OR	95% CI
Availability and use of grocery stores	Not available and/or not used	1.00		1.00	
	Available and used	1.14	0.89-1.46	0.81	0.55-1.18
Availability and use of outdoor recreational facilities	Not available and not used	1.00		1.00	
	Available and not used	1.19	0.83-1.71	1.36	0.97-1.90
	Available and used	1.09	0.87-1.37	1.17	0.89-1.55
Availability and use of social-assistance facilities	Not available and not used	1.00		1.00	
	Available and not used	**1.41**	**1.07**-**1.86**	**1.42**	**1.05**-**1.91**
	Available and used	1.09	0.85-1.38	1.24	0.94-1.64
Model 3: Availability and use of restaurants					
		Model 3A^a^ Within 400 m from home		Model 3B^a^ Within 800 m from home	
		OR	95% CI	OR	95% CI
Availability and use of restaurants	Not available and not used	1.00		1.00	
	Available and not used	**1.40**	**1.07**-**1.84**	1.04	0.69-1.58
	Available and used	**1.48**	**1.15**-**1.90**	1.06	0.71-1.60
Availability and use of outdoor recreational facilities	Not available and not used	1.00		1.00	
	Available and not used	1.14	0.79-1.65	1.34	0.95-1.88
	Available and used	1.03	0.81-1.29	1.15	0.87-1.53
Availability and use of social-assistance facilities	Not available and not used	1.00		1.00	
	Available and not used	**1.35**	**1.02**-**1.77**	**1.37**	**1.02**-**1.85**
	Available and used	1.03	0.81-1.32	1.20	0.91-1.59

Abbreviation: OR, odds ratio.

^a^ Adjusted by: schoolchildren age, schoolchildren sex, maternal education level, area level population density, and area level average income.

Bold confidence intervals mean that coefficients are statistically significant.

Having social-assistance facilities available around the home and not using them was consistently associated with higher odds of obesity in almost all models and both buffer sizes (Tables 3 and 4). For instance, in model 3A (availability and use of restaurants within 400 m buffer), children who had social-assistance facilities available and did not use them had 35% higher odds of being overweight (OR = 1.35; 95% CI = 1.02-1.77) as compared to those with no availability. Stronger associations for the social-assistance environment were found in model 1A (availability and use of snack outlets within 400 m buffer; OR = 1.42; 95% CI = 1.07-1.87) and model 2B (availability and use of grocery stores within 800 m buffer; OR = 1.42; 95% CI = 1.05-1.91).

Table 4 shows coefficients from the fully adjusted model containing snack outlets, grocery stores, and restaurants together. Results from this model were very similar to models 3A and 3B, where children with a restaurant available had 40% higher odds of being overweight (OR = 1.40; 95% CI = 1.06-1.85) when compared to those who do not have them near home or do not used them. Stronger associations between overweight and “availability and use of restaurants” were observed for those who reported to use restaurants available near home (OR = 1.48; 95% CI = 1.14-1.93) when compared to both schoolchildren who do not have them near home or do not used them and to those with a restaurant available but not used (OR = 1.40; 95% CI = 1.06-1.85). Once more we found an addition of 8% in the odds of being overweight if restaurants near are used when compared to those near home that were not used.

**Table 4.** Multivariate Analyses of Association Between Overweight and Availability and Use of a Diversity of Food Stores, Outdoor Recreational Facilities, and Social-Assistance Features Among Schoolchildren Aged 7 to 14 years Living in Florianópolis, SC, Brazil, 2012/2013.

Model 4: Availability and use of restaurants, grocery stores, snack outlets					
		Model 4A^a^ Within 400 m from home		Model 4B^a^ Within 800 m from home	
		OR	95% CI	OR	95% CI
Availability and use of restaurants	Not available and not used	1.00		1.00	
	Available and not used	**1.40**	**1.06**-**1.85**	1.02	0.66-1.58
	Available and used	**1.48**	**1.14**-**1.93**	1.04	0.68-1.60
Availability and use of grocery stores	Not available and/or not used	1.00		1.00	
	Available and used	1.09	0.84-1.41	0.76	0.52-1.13
Availability and use of snack outlets	Not available and/or not used	1.00		1.00	
	Available and used	0.95	0.73-1.23	1.19	0.85-1.67
Availability and use of outdoor recreational facilities	Not available and not used	1.00		1.00	
	Available and not used	1.14	0.79-1.64	1.35	0.96-1.90
	Available and used	1.02	0.81-1.32	1.15	0.87-1.53
Availability and use of social-assistance facilities	Not available and not used	1.00		1.00	
	Available and not used	**1.34**	**1.01**-**1.78**	**1.38**	**1.02**-**1.87**
	Available and used	1.03	0.80-1.32	1.21	0.91-1.60

Abbreviation: OR, odds ratio.

^a^ Adjusted by schoolchildren age, schoolchildren sex, maternal education level, areal level population density, and area level average income.

Bold confidence intervals mean that coefficients are statistically significant.

## Discussion

This study analyzed the combined availability and use of 3 domains of the environment (food, physical activity, and social-assistance environments) surrounding the homes of schoolchildren living in Florianópolis (Southern Brazil), in association with overweight. Greater availability of restaurants within 400 m of schoolchildren’s homes was associated with higher odds of overweight, and stronger associations were found for those reporting to use restaurants available near the home. This association was found both in models where restaurants were analyzed individually and also when adjusting for the broader food environment (ie, presence of other food outlets). Additionally, schoolchildren who had social-assistance facilities surrounding their homes but reported not to use them also showed consistently higher odds of being overweight. Facilities of the physical activity environment were not associated with overweight in the evaluated models.

An association between geographical availability of food outlets and overweight has been frequently investigated and has shown inconsistent findings.^10,11^ In this study, we found a positive association between both “availability only” and “availability and use” of restaurants and higher odds of overweight. Since food environment studies rely on the assumption that availability of a food outlet translates into use of that food outlet, one could think that when analyzing both the availability and use of food outlets, an association with overweight would be found only for “use of food outlet” and not for “availability.” It is not clear what underlying mechanisms would be involved for an association between the availability of restaurants, while not using it, and overweight. It could be that some degree of social desirability bias occurred in such way that parents of some overweight children misreported their use of restaurants, but we cannot be sure about that. Most research on social desirability bias, within this context, focused on (under)reporting of energy intake and not use of restaurants. For example, Guinn et al found that children showing higher scores of social desirability bias had lower accuracy while reporting their energy intake.^63^ This complex relationship between exposure to food outlets and its use has been highlighted by others as well.^64^ To address this gap, future research could explore the link between actual use of food outlets (by measuring daily activity patterns of participants) and reported use of food outlets, and its relation with overweight.

An association between the use of restaurants and overweight may be supported by the fact that families choose more calorically dense, highly processed foods when eating out, potentially leading to a higher energy level intake.^13^ According to the Brazilian Household Budget Survey (POF 2008-2009), only 5.7% of products purchased by Brazilian families in restaurants are fresh products such as salads and vegetables,^51^ and 18.2% are ultra-processed foods.^65^ Indeed, in a previous study with children living in Florianópolis, the use of restaurants was associated with a higher consumption of foods composing a “Fast Food” pattern.^66^ In addition, a study using data from the Brazilian Household Budget Survey (POF 2008-2009; n = 34 003 children up to 10 years old) found that a “ultra-processed food” pattern, characterized by foods such soft drinks, fast food, pizza, and sweets, was a very common choice among children when eating out. Since out-of-home meals are becoming increasingly popular in Brazil,^67^ such findings demonstrate the need for policies aiming to limit the amount of ultra-processed foods offered in restaurants, especially those near schools. Such policies are timely and essential to achieve the “Brazilian Strategic Plan to fight non-communicable diseases—2021 to 2030,” whose aims include the reduction of childhood obesity prevalence to 6.7% as well as decrease population level consumption of ultra-processed food and sweetened beverages intakes to 13% to 15%.^68^ Interestingly, the presence and use of snack outlets were not associated with being overweight in this study, as food outlets often colocate, we expected to find an association as the one found for restaurants. It is important to highlight that while the literature on the influence of snack and fast food outlets reports mixed evidence for an association between access to these retailers and obesity in children and adolescents,^27^ such international and Brazilian studies focused mostly on the availability aspect, disregarding the use of such retailers.^27,69,70^ One possible explanation for this lack of association in our study could be that dichotomizing the variable for availability and use of snack outlets led to a decrease in its variability, making it less likely to find a significant association, what means that weekly or monthly use of snack bars can be more prevalent among children and their families when compared to another facility from the food environment. This was not a limitation to the restaurant variable because a higher percentage of children reported to make frequent use of restaurants than snack outlets. Due to data distribution, we were forced into this dichotomization and consequently lost the nuanced frequency information provided by the parents on their child’s usage of facilities. Nonetheless, we acknowledge that there is likely a qualitative difference between a child who rarely uses a snack outlet in comparison to a child who uses a food outlet weekly. Indeed Emond and colleagues found a dose–response association for a more frequent intake of fast food and obesity in children. Over a period of 1 year, they found that the risk of weight gain was 38% higher by each additional weekly fast food intake.^71^

A consistent association for availability of social-assistance facilities and higher odds of overweight was found among children who reported not to use these facilities but not among children who reported to use them. Social-assistance facilities tend to be located in low socioeconomic areas.^72^ One potential explanation for this finding is that in our sample, we observed a greater concentration of children with high BMI in both highly deprived and highly affluent areas. Families of low socioeconomic position are more likely to experience inadequate access to health care,^73,74^ thus, children of low socioeconomic position may not have the structural resources needed to access social-assistance facilities (eg, lack of transportation). On the other hand, (overweight) children living in affluent areas are not targeted by the social-assistance facilities/programs and therefore are less likely to use these facilities. Another potential explanation for this finding may be that children who do not use these facilities are not reached by social programs and may not benefit from a higher social cohesion and consequently a potential healthier lifestyle.^75-77^ As suggested by previous literature, children experiencing higher social cohesion spent more minutes per day outdoors and had a higher number of walking and cycling trips per week, these behaviors and stronger social ties were associated with lower BMI.^46,58-60^

The present study found no significant associations between the availability and use of physical activity facilities and overweight. This is in accordance with previous research that analyzed aspects of the physical activity environment and overweight.^78^ Nonetheless, the usage of physical activity facilities in relation to childhood obesity has been rarely explored. Previous systematic reviews concluded that environmental characteristics such as presence of parks and playgrounds has likely a positive influence on physical activity levels.^79,80^ Given the positive impact of physical activity on children’s BMI, future research could explore whether the use of physical activity facilities is associated with overweight. Additionally, it has been suggested that participation in neighborhood initiatives could be important motivations to change health behavior, such as exercising more outdoors,^20,81^ but we could not demonstrate that. Similar to the present study, Hoenink et al when evaluating 5199 adults from 5 European countries found no significant associations between overweight and availability of recreational facilities such as gyms, swimming pools, and sports clubs. However, the perception of having social cohesion in the neighborhood had an inverse association with overweight and obesity.^81^ Furthermore, it may be that availability measures are not the most appropriate to indicate children’s exposure to the physical activity environment. As demonstrated by a systematic review that found that aggregated measures of physical activity environment (eg, walkability) were more strongly associated with weight status in children than specific measures as availability.^82^

Limitations of this study include the use of secondary data to measure the availability of food and social-assistance services facilities as well as the use of self-reported measurements to evaluate the use of food outlets. However, we used a complete method for triangulation of data sources for secondary data collection. Besides that, the use of secondary data is justifiable due to the complexity of this study that evaluated 3 domains of the built environment.^83^ The data for this study were collected in 2013, therefore, possible urban changes may have occurred in this period, requiring further investigations to update the scenario. Therefore, we emphasize that the data of the present study portray the historical context of the year 2013. Over the last 9 years, it is likely that all the variables investigated have changed. In addition to changes in economic and social factors, Brazil experienced the coronavirus disease 2019 pandemic period in the last 2 years, which undoubtedly must have changed the local context of these investigated variables on the exposure (food outlets/physical activity and social assistance places) and the outcome (BMI of schoolchildren). In addition, given the socioeconomic characteristics of the study area, our results may not be generalizable to all Brazilian regions. Nonetheless, this study has many strengths, including being one of the few studies in food environment research to analyze the influence of both the objectively measured food environment and the use of different types of food outlets. The fact that the physical activity and social-assistance environments were accounted for in the models is also a strength of this study, as most studies about environmental influences on health are limited to only one domain of the environment, for example, only the food or physical activity environments. Nonetheless, a single neighborhood construct is likely not enough to explain the influence of environmental characteristics on health outcomes, including overweight among children. Therefore, while accounting for the influence of different environmental domains, this study brings insight on how the use of environmental facilities may influence the association of some food outlets and overweight. Other strengths of this study include the strong protocol used to objectively measure overweight and obesity in the sample; the training of the data collection team; the protocol for adaptation of the survey questionnaires, which were subject to experts’ reviews; the pilot study performed before data collection; and the big sample size which was drawn from areas of diverse residential density and income and was selected by clusters according to the number of students in each school. All these points contribute to support the internal validity of our study.

## Conclusions

This study examined associations between the food, physical activity, and social-assistance environments and overweight and obesity among Brazilian schoolchildren. Greater availability of restaurants around the home was associated with higher odds of overweight, and this association was stronger for children reporting to use restaurants. Having higher availability of facilities from the social assistance environment around the home, but not using them, was associated with higher odds of overweight. Future research could also consider intermediate factors that may have an influence in the relationship between the presence and use of facilities and overweight and obesity, such as social network influences, affordability of healthy food, the understanding of the monetary value of healthy food, transportation to facilities, and targeted marketing and advertising of unhealthy foods. By using a combined measure of availability and use of food outlets, findings of the present study add to the body of evidence on obesogenic environments and highlight the need for policies that limit the access to obesogenic food outlets, especially by children and adolescents. Our results also highlight the need for more studies evaluating the influence of social-assistance facilities on overweight and obesity.

## Supplemental Material

Supplemental Material, sj-pdf-1-fnb-10.1177_03795721221146215 - Neighborhood Availability and Use of Food, Physical Activity, and Social Services Facilities in Relation to Overweight and Obesity in Children and Adolescents Click here for additional data file. Supplemental Material, sj-pdf-1-fnb-10.1177_03795721221146215 for Neighborhood Availability and Use of Food, Physical Activity, and Social Services Facilities in Relation to Overweight and Obesity in Children and Adolescents by Camila Elizandra Rossi, Maria Gabriela M. Pinho, Elizabeth Nappi Corrêa, Ângelo Horta de Abreu, Cassiano Ricardo Rech, Jorge Ricardo da Costa Ferreira and Francisco de Assis Guedes de Vasconcelos in Food and Nutrition Bulletin
