# Supplementary material for: Neighborhood Availability and Use of Food, Physical Activity, and Social Services Facilities in Relation to Overweight and Obesity in Children and Adolescents
Source: Food Nutr Bull. 2023 Jan 4;44(1):12–26. doi: 10.1177/03795721221146215 (PMC10186564; doi:10.1177/03795721221146215)
Supplement: Supplemental Material, sj-pdf-1-fnb-10.1177_03795721221146215 - Neighborhood Availability and Use of Food, Physical Activity, and Social Services Facilities in Relation to Overweight and Obesity in Children and Adolescents [file sj-pdf-1-fnb-10.1177_03795721221146215.pdf]

Supplementary Table 1. Distribution of food environment variables regarding availability and use of snack outlets and grocery stores before dichotomising the categories into 'Not available and/or not used' and 'Available and used'.

**Variables**	**Categories**	**N**	**%**
Availability and use of snack outlets– 400 m (n =1949)	Not available	513	26.3
	Available and not used	63	3.2
	Available and used	1,373	70.5
Availability and use of grocery stores – 400 m (n =1913)	Not available	498	26.0
	Available and not used	10	0.5
	Available and used	1,405	73.4
Availability and use of snack outlets – 800 m (n =1949)	Not available	178	9.1
	Available and not used	71	3.6
	Available and used	1,700	87.2
Availability and use of grocery stores – 800 m (n =1913)	Not available	128	6.7
	Available and not used	14	0.7
	Available and used	1,771	92.6

Supplementary Table 2. Spearman correlation analysis including all exposure measures calculated around 400 metres and 800 metres around the home of 7 to 14-year-old schoolchildren, Florianópolis, Santa Catarina, Brazil, 2012/2013.

	**400 meters of schoolchild's homes**				
**Availability and use of:**	**snack outlets**	**grocery stores**	**restaurants**	**outdoor recreational facilities**	**social-assistance facilities**
snack outlets	1.0000				
grocery stores	0.3823	1.0000			
restaurants	0.3506	0.2316	1.0000		
outdoor recreational facilities	0.3174	0.2865	0.3512	1.0000	
social-assistance facilities	0.3534	0.2948	0.2192	0.3246	1.0000
	**800 meters of schoolchild's homes**				
**Availability and use of:**	**snack outlets**	**grocery stores**	**restaurants**	**outdoor recreational facilities**	**social-assistance facilities**
snack outlets	1.0000				
grocery stores	0.3074	1.0000			
restaurants	0.2107	0.1302	1.0000		
outdoor recreational facilities	0.2264	0.1488	0.2415	1.0000	
social-assistance facilities	0.2429	0.1799	0.0601	0.3650	1.0000
